# Effectiveness of non-technical skills training for healthcare professionals in emergency departments: a systematic review

**DOI:** 10.1186/s13049-026-01574-9

**Published:** 2026-02-02

**Authors:** YuKun Zhang, YuWei Wang, ManYu Jiang, XiHui Sun, LiYoong Tang

**Affiliations:** 1 Department of Nursing, The Second Affiliated Hospital of Zhejiang University School of Medicine, Hangzhou, China; 2 Department of Nursing Science, Faculty of Medicine, Universiti Malaya, Kuala Lumpur, Malaysia

**Keywords:** Emergency department, Non-technical skills, Teamwork, Simulation training, Patient safety, Systematic review

## Abstract

**Objectives:**

This systematic review evaluated the effectiveness of non-technical skills (NTS) training for healthcare professionals working in emergency departments (EDs) and assessed the certainty of the evidence.

**Methods:**

Peer-reviewed studies of NTS training for ED staff, including physicians, nurses, and allied health professionals, were systematically identified from PubMed, Scopus, Web of Science, the Cochrane Library, CINAHL, EMBASE, and PsycINFO, with supplementary searches of Google Scholar and reference lists (search date: 19 August 2025). Eligible studies reported learning outcomes, observed behavioural performance, and/or patient- or process-level outcomes in ED settings. Two reviewers independently screened records, extracted data, and assessed risk of bias using RoB 2 for randomised controlled trials, ROBINS-I for non-randomised comparative studies, and the NIH/NHLBI tool for before-and-after studies. Owing to methodological heterogeneity, findings were synthesised narratively, and certainty of evidence was assessed using the GRADE approach (PROSPERO: CRD420251181995).

**Results:**

Of 6,359 records identified, 15 studies met inclusion criteria, comprising two randomised controlled trials, one quasi-experimental study with a control group, and twelve before-and-after studies, predominantly from single-centre adult EDs in North America, Europe, and Asia. NTS training was commonly delivered through simulation-based courses with structured debriefing and was associated with consistent pre- to post-training improvements in knowledge, teamwork attitudes, and self-confidence, with low to moderate certainty of evidence. Behavioural performance improved across study designs, including more frequent closed-loop communication, clearer role allocation and prioritisation, improved situational awareness, and higher teamwork ratings measured using validated instruments such as the Team Emergency Assessment Measure (TEAM) and the Trauma Non-Technical Skills scale (T-NOTECHS) (moderate certainty). Evidence for clinical or process outcomes was limited but generally favourable in single-centre studies, including shorter resuscitation times and improved protocol adherence; no study reported effects on mortality or complication rates, and the certainty of evidence for these outcomes was rated as low to very low.

**Conclusion:**

NTS training for ED teams improves learning outcomes and observable team performance, particularly when simulation is combined with high-quality debriefing and brief in-situ refreshers. Evidence for effects on patient outcomes remains limited. Future research should prioritise pragmatic multi-site designs with blinded video-based assessment and pre-specified process and patient endpoints.

**Supplementary Information:**

The online version contains supplementary material available at 10.1186/s13049-026-01574-9.

## Background

Non-technical skills (NTS) are defined as the cognitive, social, and personal resource skills that complement technical expertise and contribute to safe and efficient task performance [1, 2]. They encompass domains such as leadership, communication, situational awareness, decision-making and teamwork [3]. First developed in high-reliability industries such as aviation and nuclear power, the NTS framework has since been adapted to healthcare to address the complex, dynamic, and high-risk nature of clinical environments [4, 5]. In emergency care, NTS are recognized as an essential complement to technical proficiency, ensuring not only the accuracy of interventions but also their timeliness and safety [6].

Deficiencies in NTS have been identified as major contributors to adverse outcomes in healthcare. Multiple reports estimate that human factors account for approximately 43–70% of adverse events in emergency and operating room settings, with shortcomings in teamwork and communication identified as the root cause in up to 50% of cases [7–10]. In the emergency department (ED), where rapid decision-making, multitasking, and interprofessional coordination are essential, poor NTS have been linked to delayed diagnosis, ineffective resuscitation, communication breakdowns, and higher rates of medical error [11].

Given the time-critical and resource-constrained nature of ED work, NTS are indispensable for ensuring patient safety and optimal team performance. Skills such as effective communication, situational awareness, and leadership are vital for coordinating rapid responses to life-threatening emergencies [12, 13]. However, despite the importance of NTS for patient safety in emergency care, most systematic reviews to date have concentrated on surgical environments or intensive care units (ICUs) [14, 15]. Moreover, direct evaluation of patient-level outcomes following NTS training in emergency care remains uncommon. This reflects the inherent difficulty of attributing downstream clinical outcomes, such as mortality or complications, to educational interventions that primarily influence team cognition, coordination, and behaviour within complex clinical systems [16, 17]. Consequently, research in this field has predominantly focused on intermediate outcomes, including observable team behaviours and process measures, which are conceptually closer to the intervention and more sensitive to change. Therefore, this review systematically evaluates the effectiveness of NTS training in EDs to address a key gap in the current evidence base.

### Aims

- ❿ To provide a systematic overview of the effectiveness of NTS training programs in EDs.
- ❿ To evaluate the impact of NTS training on team performance, patient safety, and clinical outcomes in ED settings.

## Methods

### Protocol and registration

This review is registered with PROSPERO (Ref No: CRD420251181995). The report of this review followed the recommendations of the Preferred Reporting Items for Systematic Reviews and Meta-Analyses (PRISMA) guidelines [18].

### Eligibility criteria

Studies were considered eligible if they (1) involved healthcare professionals working in EDs; (2) investigated structured interventions designed to enhance NTS, including but not limited to communication, teamwork, leadership, decision-making, situation awareness, and task management; and (3) reported outcomes related to team performance, patient safety, or clinical processes. Both randomised controlled trials and quasi-experimental or pre–post intervention studies were included. Training programs delivered through approaches such as simulation, workshops, or multimodal learning formats were eligible for review.

Studies were excluded if they (1) targeted only leaders or managers rather than frontline ED staff; (2) presented training programs solely within the context of broader quality improvement initiatives without isolating the specific effects of NTS training; (3) employed observational, qualitative, pilot, validation, or case study designs, or were review articles; (4) were reported as conference abstracts, dissertations, or other unpublished grey literature; or (5) were not published in English or did not have full-text availability.

### Search strategy

Peer-reviewed studies were retrieved from seven databases: PubMed (Medline), Scopus, Web of Science, the Cochrane Library, CINAHL (EBSCOhost), EMBASE (Elsevier), and PsycINFO. Searches were restricted to studies published in English, consistent with the predefined eligibility criteria. Google Scholar was additionally searched for grey literature, and reference lists of eligible reports were screened. Searches were completed on 19 August 2025. Full search strings for each source are provided in Supplementary Material 1.

### Study screening

**Fig. 1:**
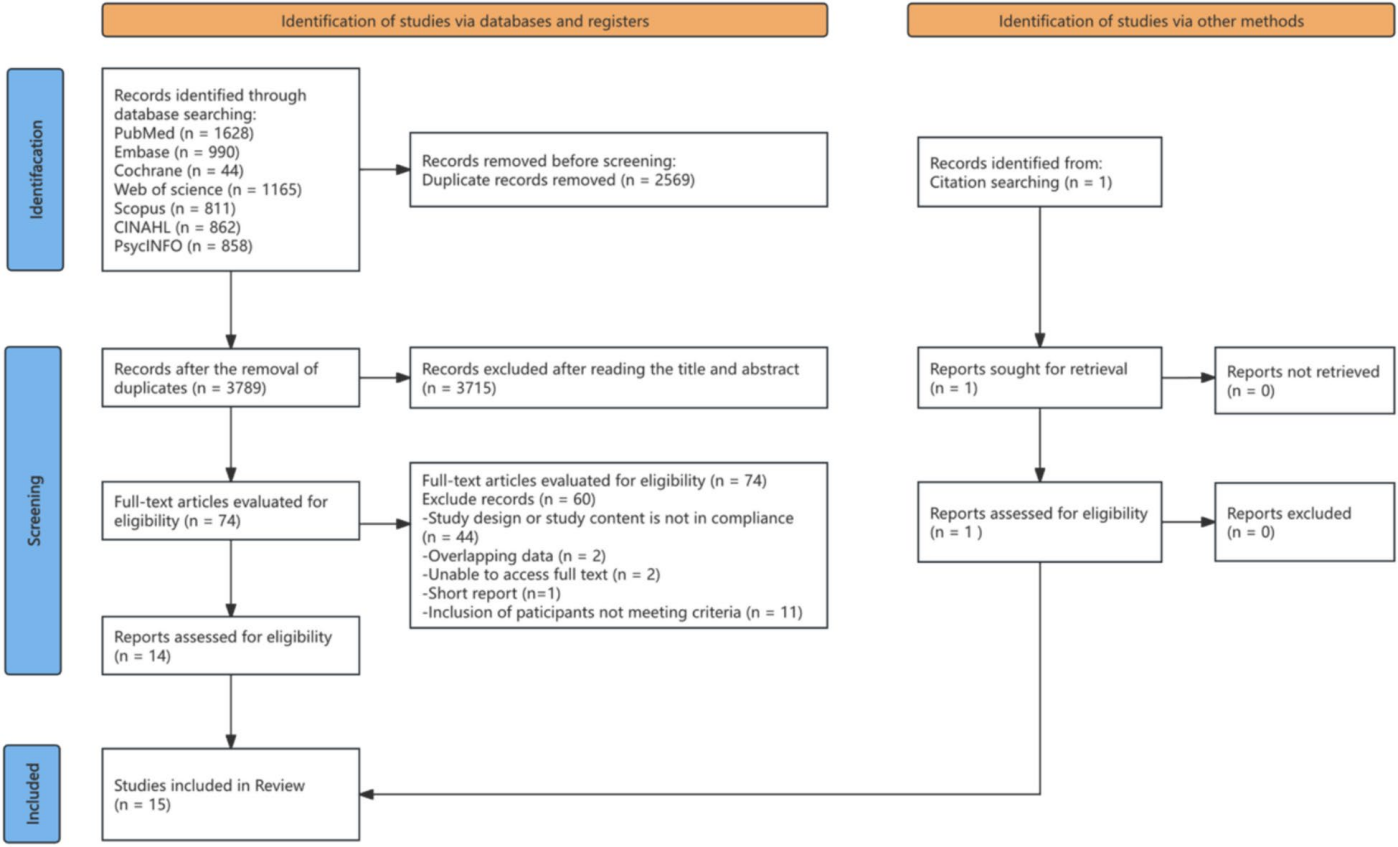
PRISMA Flow diagram of article screening and selection process

All search results were imported into EndNote 21 for reference management. Duplicate records were removed before screening. Two reviewers (M.Y.J. and X.H.S.) independently screened titles and abstracts in pairs. Articles considered potentially eligible were then assessed in full text against the predefined inclusion and exclusion criteria. Disagreements were resolved through discussion to reach consensus, and when needed were adjudicated by a third reviewer (L.Y.T.). The study selection at each stage is presented in the PRISMA flow diagram (Fig. 1).

### Data collection

Data extraction was conducted independently by two reviewers using a predesigned standardized form. The form was developed with reference to the Cochrane Effective Practice and Organization of Care (EPOC) principles and was adapted to fit the specific objectives of this review [19]. Extracted information included study characteristics (author, year, country, design, and setting), participant demographics, details of the NTS training intervention (content, duration, frequency, teaching strategies, facilitators/instructors, and co-interventions), comparator interventions, evaluation methods, and outcome measures. Discrepancies between reviewers were resolved by discussion or consultation with a third reviewer.

### Risk of bias assessment

Risk of bias was appraised according to study design. Randomised controlled trials were evaluated with the Cochrane Risk of Bias 2 (RoB 2) tool [20]. Nonrandomised comparative studies were judged using ROBINS-I [21]. Uncontrolled before-and-after studies were examined with the NIH/NHLBI Quality Assessment Tool [22]. Two reviewers conducted all ratings independently; disagreements were resolved through discussion, with a third reviewer consulted when needed. Inter-rater reliability was quantified using Cohen’s kappa. Inter-rater reliability was quantified using Cohen’s kappa, with substantial agreement between reviewers (κ = 0.78).

### Quality of evidence

The certainty of evidence was evaluated using the Grading of Recommendations, Assessment, Development and Evaluation (GRADE) approach, adapted to the context of NTS training in emergency care. For each outcome domain (teamwork performance, process/efficiency measures, patient safety indicators, and organizational outcomes), certainty was rated as high, moderate, low, or very low. Ratings considered risk of bias, coherence of findings across studies, applicability to ED settings, imprecision, and potential publication bias.

### Data synthesis

Data were synthesized narratively according to the predefined data collection plan. Summary tables were used to present study characteristics, intervention features, and reported outcomes. Given the heterogeneity in study designs, training content, outcome measures, and evaluation methods, quantitative meta-analysis was not feasible. Instead, the results were summarized descriptively, highlighting consistencies and discrepancies across studies and exploring possible explanations such as differences in training approaches, participant characteristics, and contextual factors.

## Results

Following the PRISMA process (Fig. 1), a total of 6,359 records were identified (6,358 from databases and 1 through citation searching). After removing 2,569 duplicates, 3,789 titles and abstracts were screened, and 3,715 were excluded. Seventy-four full texts were assessed for eligibility, with 60 excluded for reasons such as non-compliant study design or population. Ultimately, 14 studies from databases and 1 from citation searching were included, yielding 15 studies in the final review.

### Characteristics of studies and participants

Fifteen studies published between 2011 and 2025 were included. Most were conducted in adult EDs (14/15), with one study in a paediatric ED. Geographically, studies came from the United States (*n* = 9), Korea (*n* = 1), Canada (*n* = 1), Romania (*n* = 1), New Zealand (*n* = 1), Australia (*n* = 1), and Italy (*n* = 1). Of the 15 studies, 13 were single-centre and 2 were multicentre (one 7-site study and one 5-site study).

With respect to design, two were randomised controlled trials [23, 24], one was quasi-experimental with a control group [25], and twelve were before-and-after studies without a control group [26–37].

Participants were multidisciplinary ED personnel, including attending and resident physicians, registered nurses, respiratory therapists, technicians, pharmacists, and (in some studies) mid-level practitioners. Professional sample sizes ranged from 13 [25] to 324 [29]. Demographic reporting for professionals was limited; where available, mean ages included 27.9 ± 3.2 years [24] and 30 years [37], and male proportion ranged from 23.7% [30] to 71.4% [24].

Several studies additionally reported patient cohorts for outcome assessment, with sample sizes of 74 [32], 244 [28], 342 [23], and 650 [31]. Reported mean patient ages were approximately 39–45 years in studies that provided these data. Detailed characteristics are provided in the Additional File 2.

### Characteristics of the interventions

Training courses were predominantly short term and delivered within a single working day, with sessions lasting from 1.5 to 7 h, and one extended format consisting of a two-week course [33]. Follow up ranged from 2 to 24 months across nine studies and was primarily applied to assess behavioural or performance related outcomes [23, 24, 26, 28, 31, 34–37].

Common training themes focused on developing teamwork, communication, situational awareness, and leadership skills within emergency resuscitation teams. Additional elements such as decision making, stress management, and shared leadership were also addressed, though less frequently. Several studies further integrated non-technical skills training with broader domains including human factors, patient safety, and risk management [29, 31, 35].

The predominant instructional strategy was simulation-based learning, commonly combined with didactic lectures, interactive discussions, and structured debriefing sessions focusing on Crisis Resource Management (CRM) principles. Some studies adopted specific approaches such as script-based rehearsal [24], situation display systems for enhancing situational awareness [25], structured debriefing using the STOP5 format [34], and role play or procedural practice [30, 31]. To reinforce learning, certain interventions employed supplementary strategies including in-situ refreshers, educational newsletters, and visual reminders [35, 36].

Training delivery was primarily led by simulation-trained faculty and clinical educators from emergency medicine, trauma, or critical care departments. Several programs reported the involvement of multidisciplinary steering or implementation committees composed of physicians, nurses, and quality or education staff [29, 36]. In some cases, external providers or vendor-sponsored instructors contributed to specialized modules such as intraosseous access or Team STEPPS-based teamwork components [31].

Co-interventions were documented in five studies, including the introduction of new protocols, team meetings, refresher simulation activities, and behavioural reinforcement strategies to maintain training effects. In studies with control groups, participants typically received standard orientation programs or routine simulation sessions without exposure to the new NTS-focused curriculum [23, 25].

### Risk of bias analysis

The two randomised clinical trials [23, 24] showed some concerns mainly in the randomization process and in missing data. Risks for deviations from intended interventions and for outcome measurement were low. Both trials were judged as some concerns overall. The quasi-experimental study with a control group [25] was judged as moderate risk of bias. The main issues were confounding and selection of participants, while other domains were low to moderate risk.

For before-and-after studies without a control group, NIH appraisal indicated predominantly fair quality, with one study rated poor. Strengths included clear objectives, consistent delivery of interventions, and appropriate pre to post statistical testing. Recurrent limitations were the lack of sample size justification, incomplete reporting of loss to follow up, and limited blinded outcome assessment. Few studies reported prespecified eligibility criteria or repeated outcome measurements after the intervention, although most used valid measures and applied them consistently.

### Effect of NTS training

The outcomes of non-technical skills training were categorized according to Kirkpatrick’s four-level model [38]. None of the included studies reported participants’ reactions such as satisfaction or perceived relevance to the training (Level 1). Therefore, the summary focuses on Levels 2–4, covering learning outcomes, behavioural performance, and clinical results. Table 1 presents the synthesized effects across these levels, and detailed findings of each study are provided in Additional File 3.

**Table 1:** Summary of findings

Outcome focus	Effect	Study design	Measurement tool(s)	Evidence quality (GRADE)
Level 2 – Learning outcomes				
Knowledge of NTS domains (teamwork, communication, leadership) [29–31, 33–37]	Significant pre–post improvement in knowledge and understanding of CRM principles and teamwork concepts	Pre–post without control (7); quasi-experimental (1)	Self-report questionnaires; knowledge tests; HIRAID or ATCC checklists	Low – Moderate (downgraded for non-randomised design and self-reported measures; upgraded for consistency across studies)
Attitudes toward teamwork and safety climate [29, 30, 35–37]	Improved teamwork attitudes and safety culture; enhanced team climate scores	Quasi-experimental (1); pre–post (4)	Safety attitude and team climate surveys; post-training evaluations	Low–Moderate (downgraded for lack of control and response bias; upgraded for coherence of effects)
Self-efficacy and confidence in team roles [27, 31, 33, 34]	Greater confidence and readiness to lead or perform team tasks post-training	Pre–post without control	Confidence and self-efficacy scales; structured course evaluations	Low (downgraded for subjective outcomes and single-group designs)
Level 3 – Behavioral change				
Communication (closed-loop, information sharing, assertive communication) [23–26, 29, 32, 35, 36]	Significant improvement in closed-loop exchanges, clarity, and information flow during resuscitations	RCT (1); quasi-experimental (1); pre–post (6)	TEAM (communication subscales); T-NOTECHS; CRM behavior checklists; video-based ratings; structured debrief rubrics	Moderate (upgrade for presence of RCT and consistency; downgrade for risk of bias in pre–post designs)
Situational awareness (monitoring, updating, avoiding fixation) [25, 26, 29–31, 37]	Better global awareness, fewer fixation errors; improved shared displays of state and plans	Quasi-experimental (1); pre–post (5)	TEAM (SA items); T-NOTECHS; scenario rating forms; team situation-display logs	Low–Moderate (mostly non-randomised; upgrade for consistent direction of effect)
Leadership behaviors [23, 24, 27, 31–34]	Improved leadership initiation, role clarity, and decision-making in simulations and clinical settings	RCT (1); pre–post (5)	TEAM; structured leadership behavior checklists; video debrief ratings	Moderate (upgraded for RCT evidence and external validity; downgraded for measurement subjectivity)
Observed teamwork performance (coordination, task management) [23–29, 31, 32, 37]	Significant improvement in overall teamwork scores after training	RCT (2); quasi-experimental (1); pre–post (7)	TEAM; T-NOTECHS; CRM checklists; video-based rating forms	Moderate (upgraded for consistency and replication; downgraded for limited blinding and heterogeneity of scenarios)
Level 4 – Clinical results				
Resuscitation efficiency (total resuscitation time; time to complete primary/secondary survey) [28]	Shorter total resuscitation time (≈ 16% reduction) and faster completion of primary survey	Pre–post without control (1)	ED process timing/audit; linkage with teamwork scores	Very low – Low (downgraded for single-study evidence and lack of control; upgraded for objective measurement)
Process completeness & protocol adherence (completion of required steps; role/task completion; coordination accuracy) [25, 28]	Higher completion rates; fewer omissions; better coordination	Pre–post (1); Quasi-experimental with control (1)	Checklist adherence; scenario/trauma protocol completion; situation-display logs	Low (downgraded for small samples and indirectness)
Cognitive/safety proxies (e.g., target fixation, priority recognition) [25]	Fewer fixation errors; clearer prioritization/information updating	Quasi-experimental (1)	Observer coding; team situation-display records; structured rating forms	Low (downgraded for observational bias and imprecision)

GRADE ratings were assigned based on study design, risk of bias, consistency, directness, and precision. High: Evidence from multiple RCTs with consistent results. Moderate: Evidence from ≥ 1 RCT or consistent quasi-experimental/pre–post studies. Low: Evidence from single-group pre–post or quasi-experimental studies with potential bias. Very low: Single-study evidence or indirect outcomes

Downgrading reasons included non-randomised design, self-reported outcomes, small sample size, and measurement subjectivity. Upgrading was applied when findings were consistent across multiple studies or included RCT evidence

### Level 2 learning outcomes

Across the included studies, participants demonstrated measurable gains in knowledge of CRM principles and core NTS concepts after training, reflected in improved understanding of team roles, communication strategies, leadership priorities, and structured assessment frameworks. Significant pre–post improvements in conceptual knowledge were reported in studies by Wong (2016), Harvey (2019), Munroe (2016), and Hughes (2014), with participants showing enhanced understanding of team coordination and clinical communication following simulation-based workshops or blended courses.

Attitudinal changes were also observed. Five studies, Wong (2016), Hughes (2014), Sweeney (2014), Munroe (2016), and Innocenti (2022) reported improved perceptions of teamwork and safety climate after interprofessional programs integrating didactic teaching, scenario-based learning, and repeated in situ reinforcement.

Self-efficacy and confidence for team roles and leadership increased after short simulation courses with debriefing. Studies by Baker (2025), Harvey (2019), Armstrong (2021), and Parsons (2018) showed that participants felt more confident in leading resuscitation teams and assuming critical roles following structured leadership-focused training.

### Level 3 behavioural change

Communication performance improved across eight studies [23–26, 29, 32, 35, 36]. Participants demonstrated more frequent use of closed-loop communication, clearer information exchange, and greater assertiveness during resuscitation scenarios after CRM-oriented training that included structured debriefing and targeted feedback.

Situational awareness showed measurable gains in six studies [25, 26, 29–31, 37]. Interventions incorporating visual team displays, CRM briefings, and simulated critical events improved monitoring, anticipation, and information updating while reducing fixation errors.

Leadership behaviours were strengthened following leadership-focused curricula and shared-leadership simulations. Seven studies [23, 24, 27, 31–34] documented clearer role establishment, prioritization, and decision-making during trauma or cardiac arrest simulations.

Global teamwork performance improved in ten studies [23–29, 31, 32, 37]. These studies consistently demonstrated higher TEAM and T-NOTECHS scores, greater coordination, and better task management after structured simulation programs, including evidence from two randomised controlled trials [23, 24].

The certainty of evidence for behavioural outcomes was moderate, upgraded for inclusion of randomised and quasi-experimental designs and consistency across studies, but downgraded for limited rater blinding and potential observation bias.

### Level 4 clinical results

Three studies evaluated clinical or process-level outcomes. Steinemann (2011) reported a 16 percent reduction in total resuscitation time and faster completion of primary surveys following implementation of simulation-based team training in a trauma bay setting. Both Steinemann (2011) and Parush (2017) found higher protocol adherence and improved task coordination, suggesting more efficient multidisciplinary workflow. Parush (2017) also observed fewer fixation errors and better task prioritization, reflecting gains in cognitive safety.

No study directly evaluated mortality, complication rates, or long-term patient outcomes. The overall certainty of evidence for Level 4 outcomes was low to very low, downgraded for small sample sizes, indirectness, and lack of control groups, but upgraded for objectivity of outcome measures.

## Discussion

This systematic review indicates that structured NTS training for ED professionals produces reliable improvements in learning and behaviour, with early indications of process-level gains. Across diverse settings and study designs, programmes centred on CRM, high-fidelity simulation, and structured debriefing were consistently associated with clearer role establishment, more frequent closed-loop communication, improved prioritisation, and higher global teamwork ratings [23–25, 27–29, 31, 32, 34, 36, 37]. These convergent behavioural effects support the premise that cognitive and social resources complement technical performance in high-risk work and can be strengthened through targeted education [2].

Within the included studies, patient-level outcomes were infrequently evaluated. This likely reflects the indirect pathway through which NTS training exerts its effects, primarily by shaping team cognition, coordination, and observable behaviours, rather than acting directly on distal clinical endpoints. Instead, several studies reported improvements in operational proxies such as resuscitation time, adherence to protocols, and reductions in fixation errors [25, 28]. This distribution is consistent with a mediational pathway in which team processes link education to clinical outcomes, as established in broader patient-safety literature [16]. The findings are consistent with a conceptual pathway in which well-specified behaviours, including closed-loop communication, anticipatory updates, and explicit task delegation, respond to instruction and debriefing and are plausibly upstream of safety and timeliness.

### Contribution relative to prior literature

This review extends earlier work in three respects. First, it aggregates the most contemporary ED-specific evidence and maps effects to the Kirkpatrick framework, which clarifies that the most consistent gains occur at the learning and behaviour levels, while evidence at the clinical outcome level remains sparse. Prior reviews across acute care confirmed that simulation with debriefing improves team processes [16, 39]. The present synthesis places ED findings within that trajectory and updates them through 2025, including trials and quasi-experimental designs.

Second, the included studies reveal an evolution from isolated courses toward system-embedded learning. Interventions that combined core simulation with short in-situ refreshers, visual prompts, and structured micro-debriefs more often reported larger or more durable behavioural improvements [31, 34, 36]. This evolution reflects implementation principles that emphasise alignment with workflow, timely feedback, and reinforcement of target behaviours in context.

Third, inclusion of studies from North America, Europe, and Asia broadens generalisability beyond Anglo-centric settings [24, 25, 37]. Similar behavioural domains improved across diverse organisational cultures, which suggests that core NTS constructs are portable and can be adapted locally without loss of function [2, 40].

### Mechanisms linking education to performance

Observed effects align with established cognitive and social learning mechanisms. Simulation followed by structured debriefing strengthens shared mental models and role clarity, which reduces coordination losses and accelerates prioritisation under uncertainty [41, 42]. Situation displays and checklists operate as cognitive scaffolds that lower working-memory load, increase cue salience, and facilitate global monitoring and timely updates [25]. Brief refreshers and micro-debriefs promote retrieval practice and error-based learning that consolidate habits. This mechanism profile is consistent with the durability of behavioural change in programmes that integrated reinforcement into routine operations [34, 36].

### Methodological strengths and limitations of the evidence

The field is progressing toward more rigorous evaluative designs, although several limitations persist. Single-group pre–post evaluations predominate, which introduces risks from testing effects, regression to the mean, and observer expectancy [29, 30, 35]. Randomised and quasi-experimental designs mitigate some threats but remain vulnerable to allocation bias, selection confounding, and incomplete outcome reporting [23–25]. Measurement heterogeneity also constrains synthesis. Many studies used self-report for knowledge, attitudes, or confidence, which inflates responsiveness but weakens construct validity. Behavioural outcomes were more robust when assessed by blinded raters using validated instruments with predefined anchors, such as TEAM and T-NOTECHS, during video review [23, 24, 37]. Most studies were single-centre investigations from high-income settings with limited reporting of contextual variables such as crowding, staffing, and case mix, thereby constraining external validity.

These limitations informed conservative GRADE ratings. Emphasis is placed on directional consistency and mechanism-based interpretation rather than pooled effect estimates, which would be misleading given heterogeneity and design constraints. Future studies should directly address these weaknesses to support more robust inference and clinically meaningful effect-size estimation.

### Implications for practice

Three principles are actionable for ED leaders and educators. First, invest in debriefing quality. Facilitators require training, behavioural anchors should be explicit, and protected time should be standard. Aligning debriefing with validated domains improves measurement fidelity and rater calibration [23, 24]. Second, reinforce in the workflow. Programmes that pair core simulation with in-situ drills, cognitive aids, and visual prompts demonstrate better transfer to clinical behaviour, particularly in trauma and cardiac arrest pathways [25, 28, 36]. Third, measure what matters. Track a small set of sensitive, mechanism-linked indicators, including closed-loop exchanges, prioritisation errors, and time to critical actions, using periodic video-based audits with reliability checks. Integrating these indicators into quality dashboards supports accountability and continuous improvement [27, 31, 32].

### Implications for research

Future research should advance design, measurement, and outcome quality. For design, adopt cluster-randomised or stepped-wedge trials that balance internal validity with feasibility in dynamic ED environments and ensure allocation concealment, fidelity assessment, and transparent attrition reporting. For measurement, use blinded, video-based assessment with predefined competency anchors, rater training, and reporting of inter-rater reliability and minimal detectable change. For outcomes, predefine mechanism-linked process endpoints and, where feasible, power for patient-centred outcomes or validated safety proxies with longitudinal follow-up to examine decay and booster effects. Expansion in paediatric EDs and in low- and middle-income settings will strengthen equity and context-sensitive adaptation.

### Strengths and novelty

This review followed a registered protocol, used comprehensive multi-database searches, and applied design-specific risk-of-bias tools with outcome-specific GRADE judgements aligned to Kirkpatrick levels [18]. The novelty lies in an ED-specific and up-to-date synthesis through 2025, articulation of implementation levers that are associated with stronger behavioural change, and explicit mapping of effects across outcome levels. These contributions clarify where evidence is actionable in current practice and where rigorous trials remain necessary, particularly for patient-centred outcomes.

## Conclusion

NTS training in the ED improves what teams know and do. When embedded in workflow and reinforced at the point of care, such training can enhance resuscitation efficiency and protocol reliability. Health systems should integrate simulation-debriefing curricula, brief in-situ refreshers, and competency-based observation into standard ED education and quality frameworks. The next research frontier is to link reproducible behavioural improvements to patient-centred outcomes through pragmatic, standardised, and blinded evaluation designs.

## Supplementary Information

Additional file 1.

Additional file 2.

Additional file 3.

## Data Availability

All data generated or analysed during this study are included in this published article and its supplementary information files.

## Abbreviations

NTS: Non-technical skills
ED: Emergency Department
CRM: Crisis Resource Management
EPOC: Cochrane Effective Practice and Organization of Care
PROSPERO: International prospective register of systematic reviews
PRISMA: Preferred reporting items for systematic reviews and meta-analyses
GRADE: Grading of Recommendations, Assessment, Development, and Evaluation
