# Supplementary material for: Effectiveness of non-technical skills training for healthcare professionals in emergency departments: a systematic review
Source: Scand J Trauma Resusc Emerg Med. 2026 Feb 2;34:50. doi: 10.1186/s13049-026-01574-9 (PMC12951912; doi:10.1186/s13049-026-01574-9)
Supplement: Supplementary file 1 — Additional file 1. [file 13049_2026_1574_MOESM1_ESM.docx]

**Supplementary material--Search equations**

| **Database** | **Search equations** | **Filters** |
| --- | --- | --- |
| PubMed (Medline) | ((((Simulation Training) OR (Clinical Simulation)) OR (High Fidelity Simulation Training)) AND (((Emergency Service, Hospital) OR (Emergency Department)) OR (Emergency Room))) AND (((((((Non-Technical Skills) OR (Soft skills)) OR (Crew Resource Management)) OR (Crisis Resource Management)) OR (Teamwork)) OR (Communication)) OR (Leadership)) | Articles |
| Scopus | ( ( TITLE-ABS-KEY ( Simulation Training ) OR TITLE-ABS-KEY ( Clinical Simulation ) OR TITLE-ABS-KEY ( High Fidelity Simulation Training ) ) ) AND ( ( TITLE-ABS-KEY ( Emergency Service , Hospital ) OR TITLE-ABS-KEY ( Emergency Department ) OR TITLE-ABS-KEY ( Emergency Room ) ) ) AND ( ( TITLE-ABS-KEY ( Non-Technical Skills ) OR TITLE-ABS-KEY ( Soft skills ) OR TITLE-ABS-KEY ( Crew Resource Management ) OR TITLE-ABS-KEY ( Crisis Resource Management ) OR TITLE-ABS-KEY ( Teamwork ) OR TITLE-ABS-KEY ( Communication ) OR TITLE-ABS-KEY ( leadership ) ) ) | Articles |
| Web of Science | Non-Technical Skills OR Soft skills OR Crew Resource Management OR Crisis Resource Management OR Teamwork OR Communication OR Leadership (Topic) AND Emergency Service, Hospital OR Emergency Department OR Emergency Room (Topic) AND Simulation Training OR Clinical Simulation OR High Fidelity Simulation Training (Topic) | Articles |
| Cochrane Library | (Non-Technical Skills):ti,ab,kw OR (Soft skills):ti,ab,kw OR (Crew Resource Management):ti,ab,kw OR (Crisis Resource Management):ti,ab,kw OR ("teamwork"or "Communication" or "Leadership"):ti,ab,kw AND (Emergency Service, Hospital):ti,ab,kw OR (Emergency Department):ti,ab,kw OR (Emergency Room):ti,ab,kw AND (Simulation Training):ti,ab,kw OR (Clinical Simulation):ti,ab,kw OR (High Fidelity Simulation Training):ti,ab,kw | - |
| CINHAL (EBSCOhost) | XB (non-technical skills Soft skills or Crew Resource Management or Crisis Resource Management or Teamwork or Communication or Leadership) AND (Emergency Service, Hospital or Emergency Department or Emergency Room) AND (Simulation Training or Clinical Simulation or High Fidelity Simulation Training) | Human and English |
| EMBASE (Elsevier) | ((('non technical' AND skills OR soft) AND skills OR crew) AND ('resource'/exp OR resource) AND ('management'/exp OR management) OR 'crisis'/exp OR crisis) AND ('resource'/exp OR resource) AND ('management'/exp OR management) OR 'teamwork'/exp OR teamwork OR 'communication'/exp OR communication OR 'leadership'/exp OR leadership AND ((emergency AND service, AND hospital OR emergency) AND department OR emergency) AND room AND ((simulation AND training OR clinical) AND simulation OR high) AND fidelity AND simulation AND training | Articles and Articles in Press |
| PsycINFO | (Non-Technical Skills or Soft skills or Crew Resource Management or Crisis Resource Management or Teamwork or Communicationor Leadership) AND (Emergency Service, Hospital OR Emergency Department OR Emergency Room) AND (Simulation Training OR Clinical Simulation OR High Fidelity Simulation Training) | Articles and English |
