# Supplementary material for: Effectiveness of non-technical skills training for healthcare professionals in emergency departments: a systematic review
Source: Scand J Trauma Resusc Emerg Med. 2026 Feb 2;34:50. doi: 10.1186/s13049-026-01574-9 (PMC12951912; doi:10.1186/s13049-026-01574-9)
Supplement: Supplementary file 2 — Additional file 2. [file 13049_2026_1574_MOESM2_ESM.docx]

**Table 1 - Characteristics of the studies by methodological design.**

| **Author (Year)** | **Country** | **Setting** | | **Professionals** | | | | **Patients** | | |  |
| --- | --- | --- | --- | --- | --- | --- | --- | --- | --- | --- | --- |
|  |  | **n** | **Type of ER** | **Professional category** | **n** | **Age**  **(Mean)** | **Male (%)** | **n** | **Age**  **(Mean)** | **Male (%)** | |
|  | **Controlled randomized clinical trials** | | | | | | | | | |  |
| Fernandez, 2020 | United States | 1 | Adult | emergency medicine and general surgical residents | 60 | 30(IG) 29(CG) | 63(IG) 70(CG) | 342(Pre118, Post224) | Pre:45(IG),43(CG) Post:43(IG),43(CG) | Pre:80(IG),86(CG) Post:75(IG),74(CG) | |
| Chung, 2011 | Korea | 7 | Adult | Resident physicians and nurses | 70 | 27.9±3.2 | 71.4 | - | - | - | |
|  | **Quasi-experimental with control group** | | | | | | | | | |  |
| Parush, 2017 | Canada | 1 | Adult | attending physicians, resident physicians, nurses, and respiratory therapists | 13 | - | - | - | - | - | |
|  | **Before and after without control group** | | | | | | | | | |  |
| Truta, 2018 | Romania | 1 | Adult | attending physicians, residents, and nurses | 70 | - | 34.3 | - | - | - | |
| Armstrong, 2021 | New Zealand | 1 | Adult | Senior emergency department nurses | 15 | - | - | - | - | - | |
| Steinemann, 2011 | United States | 1 | Adult | residents, ED and trauma attending physicians, nurses, respiratory therapists, and ED technicians | 137 | - | - | 244(Pre141, Post103) | Pre38.9, Post39.7 | Pre76, Post75 | |
| Hughes, 2014 | United States | 1 | Adult | physicians,nurses | 324 | - | - | - | - | - | |
| Munroe, 2016 | Australia | 5 | Adult | Registered emergency nurses | 38 | 29.45 | 23.7 | - | - | - | |
| Harvey, 2019 | United States | 1 | Adult | General Surgery and Emergency  Medicine Resident Physicians, RNs, respiratory therapists,pharmacists, attending physicians | 32 | - | - | 650(pre260, 6-months170, 12-months 220) | - | - | |
| Huffman, 2021 | United States | 1 | Adult | general surgery and emergency medicine residents,nurses | 25 | - | - | 74 | - | - | |
| Parsons, 2018 | United States | 1 | Adult | Emergency Medicine Residents | 14 | - | - | - | - | - | |
| Baker, 2025 | United States | 1 | Children | Pediatric emergency medicine (PEM) physicians including faculty and fellows | 27 | 20–29 (4%),  30–39(59%),  40–49(11%),  50–59 (26%) | 37 | - | - | - | |
| Sweeney, 2014 | United States | 1 | Adult | attending physicians, resident physicians, midlevel practitioners (nurse practitioners and physician assistants), nurses, nursing supervisors, nursing assistants, nurse managers, and medical technicians | 213 | - | - | - | - | - | |
| Wong, 2016 | United States | 1 | Adult | nurses and resident physicians | 72 | - | - | - | - | - | |
| Innocenti, 2022 | Italy |  | Adult | Emergency Medicine residents | 76 | 30 | 44 | - | - | - | |

IG: Intervention Group, CG: Control Group

**Table 2 - Characteristics of the interventions by study design.**

| **Author (Year)** | **Training duration** | **Follow-up** | **Training Goals** | **Training strategies** | **Training facilitator/Instructor** | **Co-interventions** | **Implementation Committee** | **Control group** | **Results** |
| --- | --- | --- | --- | --- | --- | --- | --- | --- | --- |
| **Controlled randomized clinical trials** | | | | | | | | | |
| Fernandez, 2020 | 4-hours | 20 months | leadership and patient care. | Leadership role establishment;  Information sharing & team communication;  Task planning & prioritization;  Role assignment & skills assessment;  Seeking input / shared decision-making;  Barrier identification;  Didactic + simulation;  Instructor-led debriefs. | Instructors experienced in team leadership training (specific qualifications not reported) | Standard orientation to trauma team leader role (same as control) | - | review of responsibilities, administrative information, attendance policy, and available resources | - Skill transfer to clinical setting after leadership training. - Improved team leadership behaviors during live trauma resuscitations. - Indirect patient-care benefits, mediated by leadership behaviors (better coordination/communication). |
| Chung, 2011 | 90-min | 3 weeks | leadership and team performance | Script-based role play (90 min): 17-min resuscitation script*6; round-robin reading; facilitator/director explanation; around-simulator reading; read-while-enacting behaviors. | Director with CPR experience | Lecture and video on team concept alongside simulation training | - | 90-minute training including 15-min lecture (adult pulseless arrest guidelines) + 15-min team-concept video + 60-min (3 rounds) high-fidelity simulation with debriefing. | Script-based CPR team training resulted in comparable improvements in team dynamics scores. Script-based methods may be used as an adjunct to CPR team training. |
| **Quasi-experimental with control group** | | | | | | | | | |
| Parush, 2017 | not reported | - | teamwork and situational awareness | Team situation display (interventions, timeline, patient info, team/roles, vitals, key events) to enhance information sharing, situational awareness, and teamwork during ED resuscitations. | attending physicians, resident physicians, nurses, and respiratory therapists | not reported | - | Standard simulated ED resuscitation scenarios | teamwork and team communication improved, clarified communication about interventions, and reduced target fixation. |
| **Before and after without control group** | | | | | | | | | |
| Truta, 2018 | 1 day | 2 months | team performance of non-technical skills | One day CRM, lectures on errors and CRM principles, six high fidelity scenarios two trauma four medical, instructor led debriefings, focus on technical and nontechnical skills. | Two instructors with CRM background training (one doctor and one nurse) and an IT technician | - | - | - | improved nontechnical skills in interprofessional emergency team, potentially improving patient safety |
| Armstrong, 2021 | 1h 40 min | - | teamwork quality and nurse leadership skills | One hour didactic, four ten minute scenarios, structured debriefs; shared leadership CPR with senior nurse leading the algorithm to offload the emergency physician. | Senior ED nurses with >5 years experience participated; training delivered by study team including emergency medicine specialists and clinical nurse specialist | - | - | - | A short simulation training programme improved nurse leadership and teamwork performance in the emergency department; the model could be easily replicated in other departments. |
| Steinemann, 2011 | 4 hour | 6 months | team communication, coordination and clinical efficacy of trauma resuscitation | One hour web based didactic followed by three hours in situ HPS in the ED, three fifteen minute blunt traumatic shock scenarios each session, immediate video enabled debriefing using T NOTECHS to assess teamwork. | Board-certified investigators (critical care trauma surgeon and critical care specialist) conducted training, facilitation, and debriefing | Online pretest and post-test associated with the web-based didactic | attending physicians, residents, nurses, respiratory thera-  pists, and ED technicians | - | improved teamwork and clinical performance of multidisciplinary trauma teams in both simulated and actual trauma settings; improvements included increased teamwork scores, faster and more complete trauma resuscitations, and reduced ED resuscitation time by 16% |
| Hughes, 2014 | 3 hour | 3months | teamwork and communication. | Three hour trauma focused CRM classroom program with comparison video of effective and ineffective team dynamics to teach CRM principles, targeting communication, leadership, team effectiveness, situational awareness, assertive communication, and flattening hierarchy. | Emergency Medicine, Trauma Services, Nursing, Quality Management, Patient Safety, and Surgical Services; specific instructors not detailed | - | Emergency Medicine,  Trauma Services, Nursing, Quality Management, Patient Safety and Surgical Services | - | enhances team dynamics, communication, and ostensibly, patient safety. Philosophy and culture of CRM should be compulsory components of trauma programs and in resuscitation of injured patients. |
| Munroe, 2016 | 4 hours | - | quality of patient assessment and nontechnical skills including communication, decision making, task management and situational awareness. | Four hour interactive HIRAID workshop with sessions on HIRAID components, ISBAR and graded assertiveness, and applied case studies and role plays. | Experienced emergency nurses | - | - | - | Improves emergency nurses’ patient assessment and may enhance patient care; integrating HIRAID offers a systematic approach to support safer care. |
| Harvey, 2019 | 1 day | 12 months | trauma resuscitation team quality outcomes. | One day ATCC with four hour didactic on teamwork communication nurse role QI conflict resolution, trauma bay high risk procedure review, IO catheter training didactic and hands on, manikin primary and secondary survey review, ninety minute interprofessional SBT with two high stakes trauma scenarios and conflict resolution, debriefing. | Interprofessional faculty with trauma and TeamSTEPPS expertise including trauma medical director, trauma clinical nurse specialist, surgeon, emergency medicine physician, TNCC instructors, and vendor-sponsored clinical instructor for IO training | Most had prior TeamSTEPPS interprofessional simulation; annual TeamSTEPPS for General Surgery and Emergency Medicine residents. | - | - | TeamSTEPPS SBT may boost nurse confidence and teamwork; biannual training may outperform annual for sustaining improvements. |
| Huffman, 2021 | 3 hours | - | crisis resource management (CRM) , non-technical skills (NTS) | trauma resuscitation scenarios ,(CRM) focused debriefings; Just-In-Time (JIT) CRM didactic | Faculty trained in clinical skills and simulation-based teaching; embedded nurse educators; multidisciplinary resident teams | CRM-focused debriefings after each scenario; JIT CRM didactic session before one scenario | - | - | improves residents’ non-technical skills. Case repetition followed by CRM-focused debriefings outweighed the effect of a single Just-In-Time CRM didactic |
| Parsons, 2018 | 2 week | 2 week | CRM skills | 30-minute didactic lecture on CRM principles ,high-fidelity simulation scenarios on leadership, problem solving, communication, situational awareness, teamwork, and resource use | Simulation faculty as facilitators and confederates (nurses, family members, consultants) with expertise in simulation education | - | - | - | improved CRM skills among novice EM interns;  the course is feasible to incorporate into EM residency curricula and observational learning is effective in CRM training. |
| Baker, 2025 | 2hour | 2-6months | leadership performance, knowledge, comfort, and clinical practice | Hybrid CED leadership training with 28 minute module and video, in person simulations with repetitive practice and immediate feedback, 5 minute simulated CED using STOP5, STOP5 badge cards issued. | PEM faculty and fellows as participants; study investigators and trained raters for feedback and assessment; embedded participants played by PEM faculty and advanced practice providers | - | - | - | Improved leadership, knowledge, and comfort; trainees applied CED skills clinically and planned more frequent debriefing. |
| Sweeney, 2014 | 7 hours | 9months | Crew Resource Management (CRM)，communication and teamwork | high-fidelity medical simulation to teach CRM principles and Structured Patient Encounter (SPE) ;pretraining lecture, simulated clinical cases with video debriefing, CRM principles ,standardized patient communication. | Multidisciplinary emergency department staff including attending physicians, resident physicians, physician assistants, nurses, medical technicians, and secretarial staff; professional actors portrayed patients and family members | Post-training posters throughout the department and weekly e-mails reinforcing teaching points for skill retention | - | - | improves communication in the ED, both between staff members and between staff members and patients, as evidenced by significant improvements in staff perceptions of communication quality. |
| Wong, 2016 | 3 hours | 9months | attitudes and perceptions toward teamwork, communication | Three hour course with didactic on teamwork and communication, two simulations septic shock and cardiac arrest, structured debriefs ;presession team role handouts, biweekly in situ reinforcement. | Interprofessional instructor team including simulation-trained emergency medicine faculty and nursing leadership | Biweekly in situ simulations in the ED resuscitation room; monthly electronic newsletters ('Teamwork Pearls') reinforcing training content | the simulation research steering committee within the department. | - | Improved teamwork attitudes and patient safety climate in ED; enhanced teamwork and communication behaviors; positive impact on teamwork processes with potential patient safety benefits. |
| Innocenti, 2022 | Two years; 6 sessions per year | 2 years | technical (TS) and non-technical skills (NTS) | Two year high-fidelity simulation across EM topics, six sessions per year, four teams and scenarios per session, two hour CRM intro, high acuity scenarios with SimMan 3G, immediate Plus Delta Solutions debriefs. | Certified facilitators trained in simulation and debriefing; faculty members involved in briefing, rating, and scenario control | Initial 2-hour training session including lecture and role play on CRM principles; lectures on hot-topic arguments in specific content areas before simulation scenarios | - | - | Improved technical and nontechnical skills; high fidelity simulation well received and viewed as beneficial. |
