# Supplementary material for: Effectiveness of non-technical skills training for healthcare professionals in emergency departments: a systematic review
Source: Scand J Trauma Resusc Emerg Med. 2026 Feb 2;34:50. doi: 10.1186/s13049-026-01574-9 (PMC12951912; doi:10.1186/s13049-026-01574-9)
Supplement: Supplementary file 3 — Additional file 3. [file 13049_2026_1574_MOESM3_ESM.docx]

**Table 1 - Risk of bias for randomized clinical trial studies**

| Study ID | Domain 1 Randomization process | Domain 2 Deviations from interventions | Domain 3 Missing data | Domain 4 Measurement | Domain 5 Reported result | Overall bias |
| --- | --- | --- | --- | --- | --- | --- |
| Fernandez, 2020 | Some concerns | Low | Some concerns | Low | Low | Some concerns |
| Chung, 2011 | Some concerns | Low | Some concerns | Some concerns | Some concerns | Some concerns |

**Table 2 - Risk of bias for quasi-experimental studies with a control group**

| Study ID | Confounding | Selection of participants | Classification of interventions | Deviations from intended interventions | Missing data | Measurement of outcomes | Selection of reported result | Overall bias |
| --- | --- | --- | --- | --- | --- | --- | --- | --- |
| Parush, 2017 | Moderate | Moderate risk | Low risk | Low risk | Moderate | Moderate | Moderate | Moderate risk |

**Table 3 - Assessment of the methodological quality of before and after studies without a control group**

| **Criterion** | Including Studies | | | | | | | | | | | |
| --- | --- | --- | --- | --- | --- | --- | --- | --- | --- | --- | --- | --- |
|  | Truta,2018 | Armstrong, 2021 | Steinemann, 2011 | Hughes, 2014 | Munroe, 2016 | Harvey, 2019 | Huffman, 2021 | Parsons, 2018 | Baker, 2025 | Sweeney, 2014 | Wong, 2016 | Innocenti, 2022 |
| 1. Was the study question or objective clearly stated? | Yes | Yes | Yes | Yes | Yes | Yes | Yes | Yes | Yes | Yes | Yes | Yes |
| 2. Were eligibility/selection criteria for the study population prespecified and clearly described? | Yes | Yes | CD | NR | Yes | Yes | Yes | NR | NR | NR | NR | Yes |
| 3. Were the participants in the study representative of those who would be eligible for the test/service/intervention in the general or clinical population of interest? | Yes | Yes | Yes | Yes | Yes | Yes | Yes | Yes | Yes | Yes | Yes | Yes |
| 4. Were all eligible participants that met the prespecified entry criteria enrolled? | NR | CD | No | CD | NR | NR | NR | Yes | NR | NR | NR | NR |
| 5. Was the sample size sufficiently large to provide confidence in the findings? | NR | No | Yes | NR | NR | NR | NR | No | NR | NR | NR | NR |
| 6. Was the test/service/intervention clearly described and delivered consistently across the study population? | Yes | Yes | Yes | Yes | Yes | Yes | Yes | Yes | Yes | Yes | Yes | Yes |
| 7. Were the outcome measures prespecified, clearly defined, valid, reliable, and assessed consistently across all study participants? | CD | CD | Yes | Yes | CD | CD | Yes | Yes | CD | CD | Yes | Yes |
| 8. Were the people assessing the outcomes blinded to the participants' exposures/interventions? | Yes | No | No | NR | NR | NR | NR | No | Yes | NA | NA | CD |
| 9. Was the loss to follow-up after baseline 20% or less? Were those lost to follow-up accounted for in the analysis? | Yes | Yes | NA | NR | NR | NR | NR | Yes | Yes | NR | NR | NR |
| 10. Did the statistical methods examine changes in outcome measures from before to after the intervention? Were statistical tests done that provided p values for the pre-to-post changes? | Yes | Yes | Yes | Yes | Yes | Yes | Yes | Yes | Yes | Yes | Yes | Yes |
| 11. Were outcome measures of interest taken multiple times before the intervention and multiple times after the intervention (i.e., did they use an interrupted time-series design)? | No | No | Yes | No | No | Yes | No | No | No | Yes | No | Yes |
| 12. If the intervention was conducted at a group level (e.g., a whole hospital, a community, etc.) did the statistical analysis take into account the use of individual-level data to determine effects at the group level? | NA | NA | No | NA | NA | NA | NA | NA | NA | NA | NA | NA |
| Quality Rating | Fair | Fair | Poor | Fair | Fair | Fair | Fair | Fair | Fair | Fair | Fair | Fair |

*CD, cannot determine; NA, not applicable; NR, not reported
